# The impact of nanomaterials on energy-centric form-finding of educational buildings in semi-arid climate

**DOI:** 10.1016/j.heliyon.2024.e39882

**Published:** 2024-11-04

**Authors:** Hannaneh Asgari, Samaneh Taghdir, Rezvaneh Amrollahi, Zahra Barzegar

**Affiliations:** a School of Architecture and Environmental Design, Iran University of Science and Technology, Tehran, 16846, Iran; b School of Physics Iran University of Science a and Technology, Tehran, 16846, Iran; c Environmental Studies, Tehran Urban Research and Planning Centre (TURPC), Tehran, 1964635611, Iran

**Keywords:** Form-finding, Self-cleaning nanomaterials, Energy consumption, Solar radiation, Educational building

## Abstract

In the modern world, the use of novel technologies in architecture has become highly significant and transformative for human-environment interactions. One of the most critical concerns in architecture is achieving optimal forms and selecting suitable materials for effective design across diverse climatic zones. Also, adopting innovative climate design methods in public spaces, such as educational buildings, is essential due to their strategic urban locations and diverse user populations. Therefore, the research conducted in this study focuses on two main aspects: optimizing building form based on energy consumption and solar radiation received by vertical surfaces, and, selecting appropriate nanomaterials for building surface to reduce energy usage and maintenance costs. This study begins with theoretical foundations, defining the key terms through a comprehensive review of relevant literature. Then, four identical classroom modules with consistent height and floor levels are proposed, and Energy Plus software is used to evaluate the energy consumption based on a module simulation in initial forms of square and rectangle with varying proportions. The best module and orientation is determined using specified climatic data of the coldest and the hottest days of the year. Further, investigations involve combining these modules in various layouts, emphasizing those that align with the functional requirements of educational spaces. Finally, two parameters of energy consumption and solar radiation on vertical surfaces are measured during specified time interval between sunrise and sunset. The results indicate that among the four proposed modules, the 18 × 18 module with a north-south orientation is the most optimal in the semi-arid climate of Tehran, and therefore, Type 3 layout demonstrates the best performance for energy consumption. This is while by incorporating selected nanotechnology (self-cleaning nanomaterial paint), energy usage decreases in all layouts, regardless of the season.

## 1 Introduction

Form Finding: In the realm of modern architecture, the integration of cutting-edge technological advancements has become paramount to achieve the optimal climate-responsive building forms. Optimization, a methodology that effectively integrates form generation, performance simulation, and evaluation, has emerged as a pivotal tool in this endeavor [1]. Form-Finding, defined as a design approach that generates forms based on a set of rules or algorithms derived from mathematical tools like Processing, Rhino, Grasshopper, and other scripting platforms, has gained significant traction. Accordingly, Architectural form has always been intrinsically linked to climatic conditions and available energy resources, influenced by factors such as solar radiation, orientation, wind direction, etc. Architectural form-finding encompasses a diverse range of approaches, categorized based on nature, context, and function, with solar form-finding emerging as a prominent sub-branch [2]. Climate-responsive form design plays a pivotal role in shaping the indoor environmental quality (IEQ) of buildings, influencing a wide range of factors that contribute to the overall well-being of occupants. These factors include: air, visual and noise pollution, odor, Lighting, air quality and ventilation, thermal comfort, chemical pollution [3], and, energy consumption.

Energy conservation and emission reduction have become a consensus for the sustainable development of humanity [4]. Energy stands as one of the most critical challenges in the 21st century. The twin threats of dwindling energy resources and escalating consumption have amplified the urgency of this issue. Energy consumption encompasses a broad spectrum of strategies, ranging from cutting-edge technological advancements to behavioral changes. These strategies can be categorized into various subfields, such as Nanotechnology, Natural Gas Consumption Optimization, Floor Heating Systems and Building Energy Management Systems (BEMS). Despite the abundance of energy resources in Iran, particularly fossil fuels, the country faces unique challenges in addressing energy conservation. The relatively low cost of energy has historically discouraged consumers, particularly industrial managers, from adopting energy-efficient practices. However, significant strides have been made in recent years to promote energy conservation in Iran. The development of energy efficiency standards and regulations, along with awareness campaigns and incentives, has contributed to a growing understanding of the importance of energy conservation. According to available data, Iran's energy consumption in 2013 stood at five times the global average and thirteen times that of China. These figures highlight the urgency of implementing comprehensive energy conservation strategies in Iran [5]. Therefore, the energy crisis demands immediate attention and concerted action.

Reducing energy consumption in the building sector is crucial for environmental sustainability. Researchers worldwide are continuously exploring intelligent strategies to predict building energy consumption and enhance energy performance using various dynamic methods. Building energy consumption is significantly influenced by four key factors: Building Characteristics, Occupant Behavior, System Efficiency, Performance and Climate Conditions [6]. Additionally, Energy-efficient architecture encompasses various strategies aimed at energy loss and energy gain within buildings. These strategies include defining techniques such as optimal orientation, shape, compactness, window-to-wall ratio (WWR), etc. These techniques play a significant role in determining the overall energy performance of a building, impacting both energy consumption and energy saving [7]. In this context, educational complex, as ubiquitous and influential structures, holds a fundamental role in promoting energy conservation and resource management. They serve as exemplars for sustainable practices and emphasize the importance of sustainability [8].

P. Redweik et al., emphasized the importance of paying attention to solar radiation in buildings and concluded that the radiation on the facades is less than on the roofs It is expected, but given the large areas involved, facades have a significant impact on the solar potential of buildings in an urban area [9]. Solar radiation, along with energy consumption, is one of the essential factors in building design. Most researchers believe that sunlight plays a positive role in improving comfort, user health, and energy performance [10]. As solar radiation is the primary determinant of the climate in each region, optimal building orientation for harnessing solar energy is a critical factor in architectural design. The intensity of solar radiation varies across different building surfaces, necessitating careful building orientation to maximize solar heat gain during winter and minimize it during summer [11]. Therefore, by considering energy consumption alongside receiving solar radiation, the educational building can be steered toward energy-efficient architecture.

Self-cleaning Nanomaterials: The form and materials of buildings significantly impact energy consumption and received radiation. Nanotechnology, has the capability to produce new materials, tools and systems at the molecular and atomic levels. The properties that emerge at these levels provide solutions for sustainability, and environmental preservation for future generations. Nanotechnology has transformed other technologies and significantly impacted the quality of life in various societies. This trend, has led to achievements such as reduced maintenance, lower production costs, energy efficiency, and longer lifespan [5]. Nanoarchitecture (Nano + Architecture), as a new style of contemporary architecture in the 21st century, will revolutionize the world of architecture. It influences the mindset of architects and inspires new ideas, especially in the realm of innovative building materials. Also, it has proven to be beneficial for construction (Table 1a) [12]. Nanotechnology contributes to the construction industry in two main ways; first, it optimizes and enhances the performance of existing technologies. Second, it introduces a new group of materials and products that didn't exist before the emergence of nanotechnology. Consequently, building maintenance and its components become essential activities that architects and building users must address after construction. This issue is particularly important for exposed surfaces, such as building facades. Taqia Rahman et al. reviews durability aspects of heat-reflective pavement coatings, focusing on methods to assess durability, the effects of durability on optical performance and temperature reduction efficiency, and the literature on developing durable coatings to extend lifespan [13]. Therefore, self-cleaning coatings, powered by sunlight, are the best type of coverage for urban areas, as they enable the breakdown of urban pollutants [14]. These surfaces were investigated in the 1970s by the botanist Wilhelm Barthlott, who conducted pioneering research at the University of Heidelberg on the lotus effect, which inspired the development of self-cleaning coatings (Table 1b) [15]. Self-cleaning Nanomaterials have drawn significant attention due to their unique properties and practical applications in the fields of energy and the environment. This technology, offers various advantages, including reduced repair and maintenance costs, elimination of labor-intensive efforts, and decreased time spent on cleaning [16]. Self-cleaning products are commercially available to protect surfaces against soiling and avoid the high consumption of energy and chemical detergents necessary for cleaning [17]. The performance of these surfaces depends on the angle of water contact on the surface and can be categorized into three types: hydrophobic, hydrophilic, and photocatalytic (Table 1c) [14]. Photocatalytic self-cleaning is one of the most widely used nanotechnologies in construction [15]. In the field of building materials, the semiconductor titanium dioxide (TiO_2_) is one of the most widely used and efficient photo catalysts for self-cleaning applications [17]. Its improved performance occurs only when exposed to ultraviolet (UV) light corresponding to the energy band gap. The UV light can excite electrons from the valence band to the conduction band, initiating a photocatalytic reaction. Due to its photocatalytic activity, this material possesses self-cleaning capabilities, making it suitable as a protective coating for buildings. Its, primary effect is significantly reducing the adhesion of dirt on surfaces (Table 1d) [18]. In fact, the presence of such surfaces optimizes the indirect costs associated with repair and maintenance over the building's lifespan, ensuring easy and cost-effective access to these surfaces. Additionally, self-cleaning materials have gained widespread acceptance even in historical structures. The Polluted operational environments, such as urban or industrial areas, lead to rapid dirt accumulation, which in turn raises concerns about the durability and aesthetics of buildings. Therefore, maintaining facade materials under relatively clean conditions is essential for preserving the long-term performance of coating elements [19]. The invention of photo catalyst materials had significant effects since the early 1970s. It was first utilized in the Jubilee Church of Rome in 1996. Consequently, among the widely use of these materials in construction, titanium dioxide-containing paints was also used for urban facades [20]. It is important to note that Nanotechnology can not only exhibit self-cleaning properties, but also have climatic performance. By maintaining high albedo and low emissivity surfaces, the amount of solar radiation absorbed by building envelopes is controlled and energy consumption is moderated.

**Table 1:** a) Nanomaterials in the construction industry [21] b) Self-cleaning function of lotus leaf from a microscopic view [15] c) Angle of liquid and surface in self-cleaning phenomenon [22] d) Photocatalytic phenomenon [23].

	Nano products in the building	Nano coatings	TiO_2_	Colors
a	Solar Cells Anti-Reflective Glasses Smart Windows Anti-Dust Flooring Fireproof Door and Window Self-Cleaning Surfaces Anti-Bacterial Surfaces Anti-Finger Surface	Anti-Fingerprint Easy to Clean Self-Cleaning Anti-Kettle Anti-Scratch Photocatalytic Electromagnetic radiation UV Protected Colors Switchable Covers Electrically Conductive Self-Healing Nano Primer for Anti-Corrosion Thermal Insulation	Cement Concert Glass Color Plaster Wood Composite Prefab Panels	Paramagnetic Anti-Forgery Cholesteric Self-Healing Luminous Heat Resistance Anti-Fungal Radiative Cooling Anti-Slip Solar Thermochromics Acoustic Heating Conductive Flop Effect Traffic Magnetic
b			d	
c				

According to the International Energy Agency's report in 2021, global energy consumption is projected to increase by 37 % between 2013 and 2040. The educational buildings have the highest energy consumption after residential and office buildings [8]. In this context, promoting awareness and education about energy reduction becomes crucial, especially within schools. The primary factor in climate-responsive design is optimizing the building's form and orientation, particularly considering the received amount of solar radiation. This article investigates the relationship between optimal climatic form and nanotechnology in educational buildings within Iran's semi-arid climate. The form optimization process was examined from two perspectives: energy consumption due to solar radiation on vertical building surfaces, and, the impact of self-cleaning nanomaterials, specifically titanium dioxide, on energy consumption and received radiation. The research method involves calculating and optimizing the initial proposed energy consumption and radiation for classroom modules. Subsequently, different arrangements of adjacent modules were evaluated, and the desired layout for educational buildings was determined using software calculations. The study period corresponds to the coldest and hottest days of the year.

According to the research records, the study initially addressed independent climate optimization and its influential factors. This was followed by an examination of energy consumption reduction in buildings, emphasizing the necessity of such measures in public buildings, particularly educational facilities. Additionally, relevant studies on the effects of radiation on building envelopes and the use of novel building materials, such as self-cleaning nanomaterials, were thoroughly reviewed. These investigations revealed a lack of comprehensive research with the specified objectives.

Therefore, this study conducted multidimensional and practical research in the fields of climatic form optimization, energy reduction, and the utilization of innovative materials in external building walls. Unlike previous studies with singular objectives, this research adopts a cohesive approach to climate-oriented building form-finding. The primary goals include reducing energy consumption and optimizing solar radiation reception in building design. The study also explores modular design, incorporating novel materials in the external façade to enhance material lifespan and reduce maintenance costs. By aligning the proposed approach with specific geographical and functional contexts, this research contributes to the advancement of sustainable building practices.

## 2 Literature review

In alignment with the aims of the present research, the literature review is structured into two sections: form-finding and nanotechnology. The comprehensive examination of the existing literature on optimal building form reveals a multifaceted relationship between building form and energy consumption, encompassing three distinct categories: Building Orientation and Density in Urban Blocks [24]. Comparative Analysis of Building Form's Impact on Energy Consumption “and Modular Layout Optimization for Energy Consumption Estimation [25]. Following the energy consumption research in public and high-traffic buildings, particular attention is given to educational facilities. Zhou et al. proposed a novel optimization method based on the modified competitive swarm algorithm (MCSA) to determine optimal building forms for maximizing solar radiation absorption in winter and minimizing absorption in summer. The method considers geographical latitude and identifies optimal building forms for different regions. The results indicate that the aspect ratio and number of floors significantly impact the optimal form. Alembic-shaped forms were found to perform better in terms of winter solar radiation absorption for a latitude of 48° and summer absorption for a latitude of 33°. Additionally, increasing the latitude from 33 to 43° reduced the optimal aspect ratio for north-south oriented terraces and slabs in winter [26].

In the following, Wei et al. aimed to determine the optimal block size and height for maximizing annual solar radiation absorption while minimizing building volume. Solar radiation was analyzed for 34 typical urban block patterns categorized into five groups: tower-slab, courtyard, mixed, and perimeter. The optimization process employed the NSGA-II and ANN algorithms. The results revealed the superior performance of the ANN method in identifying optimal block sizes and heights. The ideal block size was found to be approximately 100 m by 100 m, and the optimal building height ranged from 10 to 15 m [27]. Similarly, Louie et al. proposed a multi-objective optimization framework for the early stages of the urban design of residential blocks in Jinhua City, China. The framework aimed to optimize block morphology for energy efficiency, photovoltaic potential, and daylight hours. The optimization process utilized the Ladybug, Wallaby, Grasshopper, and Rhino platforms to generate 1896 valid solutions, including 58 Pareto solutions. Results demonstrated that the average Pareto solution achieved a 1.5 % reduction in energy consumption, a 52.7 % increase in photovoltaic potential, and a 50 % increase in daylight hours. Li et al. also employed data mining techniques to identify the key morphological factors influencing energy consumption in residential and public buildings. The analysis utilized data from 539 residential buildings and 153 public buildings in China. The results revealed that building orientation, shape factor, and surface-to-volume ratio were the primary morphological factors affecting building energy consumption [28]. Also, Ben Gromyko emphasized the importance of building orientation for optimizing energy consumption. Building orientation can lead to significant long-term energy and cost savings for both building owners and occupants [29].

The benefits of building optimal orientation include: Cost-effectiveness applicable during the initial design stages, Reduced energy demand, decreased reliance on more complex static systems, Enhanced efficiency of other static techniques, Improved daylight quality, reduced need for artificial lighting, and decreased internal building heating load and enhanced performance of solar controllers [30].

Similarly, Kabousová et al. proposed a climate-responsive optimization approach to balance architectural intent with climate impacts (particularly solar radiation and wind effects) as a sustainable design strategy in Slovakia. The research methodology utilized Ladybug along with the Galapagos optimization plugin in Grasshopper to simulate wind comfort for outdoor pedestrian seating. The results showed an improvement in optimal daylight hours (25 % increase in sunlight on the winter solstice) [31].

Based on research conducted in Iran, Qobadian identified the east-west orientation as the most optimal for buildings in Iran, emphasizing that the length of the building should be oriented towards the south to maximize solar energy gain in winter. Additionally; east and west facades, which receive significant energy in summer, should be minimized and well-protected by adjacent blocks and vegetation. Continuing, Fallahtafti and Mahdavinejad optimized building orientation in Tehran and investigated the impact of shape, relative compactness (RC), and glazing percentage on optimal orientation. They analyzed a set of 8 different modules with 360-degree orientations, RCs, and glazing percentages (25 %, 50 %, and 75 %). The results indicated that the optimal orientation of a building in Tehran is highly dependent on its passive solar heating potential, and the orientation and position of these modules within the building. It also highlights the role of the glazing percentage as the most significant role in determining building orientation [7]. Similarly, Mahdavinejad et al. investigated the orientation of high-rise buildings with the aim of optimizing energy consumption in Tehran. They concluded that cubic buildings are more energy-efficient, and the optimal orientation for maximizing energy gain is north-south and north-east to south-west [32]. Accordingly, Akbari and Hosseininejad found that the highest annual energy intake in Tehran occurs on the 150-degree southeast and west surfaces, while the lowest annual energy intake occurs on the north-facing surfaces. The best orientation for single-sided buildings is 180° south, the best orientation for double-sided buildings is north-south, and for four-sided buildings, the orientations are 0, 180, 90, and −90° [25]. Jahanbakhsh et al. investigated the impact of proper building orientation on reducing building energy consumption in Isfahan. Using Design Builder software, they analyzed orientations of 0–90 and 360-270°. Results revealed that the higher the intensity of radiation and the closer the angle of radiation to the vertical surface, the greater the amount of radiation received and, consequently, the heat generated on the surface [29]. In another category**,** Bennica et al. aimed to find the optimal building orientation for energy optimization and minimizing cooling and heating needs by evaluating four different residential complex forms in Brazil. The research results, using the genetic algorithm and thermal behavior simulation in Energy Plus software, demonstrated that optimal form and orientation can reduce energy demand by up to 4 % for the H-shaped form and 22 % for linear buildings [33]. Similarly, Sabah Hasab et al. analyzed the environmental performance and sustainable architecture of a 10-story building in Iraq, aiming to determine the optimal form and orientation for minimum energy consumption. They proposed five building form models in 16 orientations. Building Information Modeling (BIM) and energy consumption simulation over a 12-month period revealed that the T-shaped model has the lowest energy consumption at a rotation angle of 285° [34]. Additionally, Al Deed et al. investigated the impact of optimal building orientation on reducing energy consumption by examining passive solar architectural strategies such as form adjustments, building orientation, and thermal insulation to improve user thermal comfort and energy optimization. Eight building forms (square, rectangle, L-shaped, U-shaped and H-shaped) were simulated in three desert cities (Jeddah, Cairo, and Alexandria) and one temperate city (Berlin) using Energy Plus and Design Builder software. The results showed that the importance of optimization in desert cities is significantly greater than in temperate cities [35]. Conversely, Rui Sun et al. optimized thermal comfort based on a genetic algorithm in the early design stages of a kindergarten in China. They found that the type of building forms and the surrounding environment affect outdoor thermal comfort and reduced the overall thermal stress in Tianjin from 6.53 to 5.37 °C and in Shanghai from 3.57 to 2.87 °C [36]. Similarly, Wei Chen et al. proposed four different concept designs to demonstrate the benefits of integrating cooling systems into form optimization during the early design stages of high-rise buildings in Singapore. The results showed that an efficient cooling system can effectively minimize cooling energy consumption while allowing for good daylight performance [37]. In another perspective, Shahi et al. combined some approaches with a novel methodology to generate modular design options for extending existing buildings. The method is based on key architectural design criteria such as energy use, daylighting, life cycle impact, life cycle cost, and structural complexity, resulting in a set of Pareto-optimal design options for further evaluation and design development [38]. Also, Rahimi Far et al. aimed to reduce energy consumption by investigating the impact of building volume porosity on cooling and heating load using a parametric generative model under the influence of the percentage of surface radiation in Tehran. The process was conducted on two specific dates, the summer solstice and winter solstice. Finally, considering the higher demand for heating systems compared to cooling in Tehran, a set of quantitative recommendations based on the most optimal solution is provided that can be used as form-finding rules in the early design stages [39].

The performance gap in an educational building is greater than that in residential, administrative, and retail buildings. However, very few studies have been conducted to understand energy consumption patterns in educational buildings. A Canadian educational institution used data mining techniques and identified energy waste patterns for a complete academic year in three classrooms. The results showed that 70 % of the wasted energy is recoverable [6]. In the same vein, Atiya et al. evaluated the intensity and energy breakdown of 30 zero-energy schools in Belgium over a 4-year period. Two representative reference models were calibrated. The results demonstrated that both reference models have good credibility in assessing the energy performance of schools and are reliable and compatible for use by building modelers and energy experts in the future [40].

Furthermore, Boudayi and Hoseini et al. quantified the energy savings resulting from daylight contribution through developed case studies. When analyzing a building located in the Midwest of Brazil, in addition to proposing lamp replacement, they suggested a set of socio-educational measures, most of which involve attitudes aimed to maximize the use of daylight, leading to lower energy consumption. Similarly, Duarte et al. reduced energy consumption while maintaining thermal and visual comfort in two classrooms in Brazil by utilizing natural light and ventilation in their article. The research results, using the genetic algorithm and thermal behavior simulation in Energy Plus software, demonstrated that optimal form and orientation can reduce energy demand by up to 4 % for the H-shaped form and 22 % for linear buildings [41]. Additionally, Alam et al. reported energy consumption in buildings to be an average of 2.5 times higher than predicted. Furthermore, the results from the analysis of a 5-star Green Star-rated educational building in Melbourne, Australia, indicated performance gaps of 2.4 and 3.1 times higher for electricity and gas, respectively. Energy consumption hour in this study was 48 % of the total energy consumption over the one-year study period, which was very high and the potential source of waste. It was also noted that even though the building was empty on vacations, the mechanical system and socket loads continued operating on a weekday schedule, leading to significant energy loss. Several recommendations were provided as the conclusion of this study to minimize the performance gap in the studied educational building [6]. In another study, Khani et al. aimed to develop a multi-objective approach to optimize classrooms in the hot and humid climate of Qeshm Island. They found that variables such as window characteristics, shading devices, and, building orientation can reduce energy consumption from cooling, heating, and, lighting. These factors can also increase daylight factors, which are essential for providing visual comfort [42]. Similarly, Darvishi estimated the impact of evaluating the initial design of an open-plan school building in eight morphologies on energy performance in four climatic regions of Southeast Europe. They concluded that the method's effectiveness in reducing annual energy demand by a maximum of 35 % and increasing thermal comfort in classrooms by a maximum of 1.21 °C is better for compact and linear typologies than for other studied typologies [43]. Additionally, Azmati and Hoseini also emphasized the importance of building orientation in terms of heating and cooling loads [29]. In the context of other public building energy use, Corten et al. used regression analysis to study the energy-saving potential of HVAC system performance. They found that 7–13 % of heating energy and 41–70 % of cooling energy could be saved in the studied office and nursing home buildings [6].

Relevant research including similar cases, their simulation methods, techniques, and, research findings is listed and presented in Table 2.

**Table 2:** Previous research in the field of optimal form finding.

#	Author	Method	Case study	Findings
1	Benincá, L et al., 2023 [33]	Sketch up _ Energy plus_NSGA-II MOPSO	Residential complex in Brazil	Among the proposed building forms, Form H emerged as the most suitable option for optimizing building orientation, energy efficiency, and thermal comfort, thereby minimizing cooling and heating demand.
2	De Luca et al., 2023 [44]	Rhino_Grasshopper_ Ladybug Plugin	_	Development of Advanced computational methods were employed to generate climate-specific forms and integrate relevant criteria.
3	Liu, K. et al. et al., 2023 [45]	Grasshopper_ Ladybag, Wallacei & Honeybee Plugin	Residential complex in China	Urban form design with minimal energy consumption, maximum solar potential, and daylight hours were demonstrated. For block 15, which faces south at a 15-degree angle towards the west, open spaces are predominantly located in the center of the block.
4	Veisi,O et al. , 2022 [27]	Grasshopper	Neighborhood unit in Kermanshah, Iran	The annual solar radiation calculation in urban blocks, considering the relationship between average height and solar radiation using a genetic algorithm, demonstrates that optimal energy reception design improves energy efficiency by 3–5%.
5	Renuka, S.M. et al., 2022 [46]	BEopt plugin in Energy Plus	India; Mumbai, Shillong, Delhi, Chennai	Some of the parameters affecting building energy consumption, such as height, neighboring structures, roof materials, window area, and window material, were analyzed in all four geographical directions. The results indicate that the optimized parameters, considering their location and orientation, can reduce building energy consumption.
6	Mangan,S.D. et al., 2021 [47]	Meteonorm Calculations	Residential complex in Istanbul, Lund, Sweden	Climate variables, including humidity, wind speed, and urban heat island effect, significantly impact building performance and CO_2_ emissions. Parameters such as the number of floors, height-to-width ratio (H/W), and building orientation play a crucial role in energy consumption.
7	Bahgat, R. et al., 2020 [48]	Environment	Urban design and tourist resorts in Egypt	The results of investigating the impact of urban configurations on reducing solar radiation from neighboring building facades in warm and arid climates revealed that building orientation contributes to cooling loads reduction. Changing height-to-width ratios in linear configurations and altering width-to-length ratios in clustered configurations had the maximum effect.
8	Wang, L. et al., 2019 [49]	Rhino, Grasshopper	china	Evolutionary optimization based on building mass performance enables significant differentiation in design and facilitates explicit trade-offs and performance compromises for mass production of buildings.
9	Chen, K. et al., 2018 [37]	Energyplus, Grasshopper, NSGAII algorithm	Singapore	An investigation of 22 parametric building forms with varying courtyard sizes, was conducted to achieve better trade-offs between cooling energy consumption and daylight illumination.
10	Zhang et al., 2016 [10]	Galapagos, Octopus, Ladybug plugins, Energyplus, Genetic algorithm	Residential complex in China	Optimizing free-form buildings in cold climate regions requires achieving a balance among three objectives: increasing solar radiation, shaping coefficient, and spatial efficiency.
11	Okeil, A. et al., 2010 [50]	City shadow, Environment	Residential units	By examining the forms of urban blocks to maximize solar exposure on facades while minimizing it on roofs and neighboring buildings, strategies have been introduced to reduce urban heat islands through increased airflow.
12	Konis,K.et al., 2016 [51]	Galapagos, Octopus, and Honeybee plugins _Energy plus	Office building in Helsinki/NewYork/LosAngeles/Mexico	Using passive strategies to enhance daylight and ventilation improves daylight performance according to the Ashrae standard and PPOF validation. The SUDI ranges from 27 % to 65 %, and energy consumption varies between 4 % and 17 %
13	El-Deed et al., 2015 [35]	Design builder, Energy plus	Residential complex in Berlin - Jeddah - Cairo - Alexandria	By examining different orientations of buildings, the most efficient forms were square shapes in Berlin and rectangular shapes in Alexandria. The addition of an insulation layer to external walls led to fundamental changes in energy consumption for all building types.
14	Murgul, V.et al., 2015 [52]	Passive solar energy	Green house in Spain	Passive design based on climate considerations for fuel-free heating and cooling utilizes free solar energy.
15	Caruso, G. et al., 2013 [53]	Modeling method	China	An investigation of optimal building forms aimed at reducing direct solar radiation without compromising annual heating revealed that a compact form performs better. Rectangular plans absorb light by 20 %.
16	Mahdavinejad, M. et al., 2012 [32]	Modelling & Simulation method	High-rise building in Tehran, Iran	The cube-shaped form receives the highest energy intake. In stairwell-type buildings oriented in the S-N and NE-WS directions, maximum energy capacity can be achieved during the day.
17	Zerefos, S.C. et al., 2012 [54]	CFD Winair	Villa in the Mediterranean	By analyzing the energy consumption of buildings with polygonal and rectangular forms in the Mediterranean climate, it was demonstrated that mansard-shaped buildings in their southern orientation have a more expansive wind-sheltered space than rectangular buildings.
18	vanEsch, M.M.E. et al., 2012 [55]	Trigonometric equations, TRNSYS	Residential complex in New Zealand	Urban design parameters for appropriate orientation and effective utilization of passive solar heating potential during three months revealed the following: Street orientation impacts daily and seasonal street irradiance patterns and thermal comfort in different seasons. Wider streets lead to increased solar radiation efficiency.
19	Kämpf, J.H. et al., 2010 [56]	Radiance, evolutionary algorithm	Urban design in Switzerland	The results of multi-objective optimization in three urban forms with maximum solar radiation on facades demonstrated that rational utilization of solar radiation reduces energy demand for heating, cooling, artificial lighting, and costs.

Photocatalytic materials possess a wide range of desirable properties, including self-cleaning capabilities and the ability to transform pollutants into harmless substances. These characteristics make them valuable tools for pollution control, air quality improvement, and cost reduction strategies. Dionysios and Sourkouni explored recent advancements in titanium dioxide (TiO₂) photo catalysts, highlighting their extensive applications in energy and environmental fields. Their study specifically analyzed the critical role of radiation and water as essential components of the photocatalytic process. Additionally, Andaloro et al. addressed the importance of maintaining and cleaning building facades due to their high cost. They proposed that the application of nano-coatings on building exteriors can significantly facilitate the cleaning process. Also, several studies have explored the impact of building design on energy efficiency and solar radiation management. For instance, Habib Mansour et al. discussed sustainable self-cleaning treatments for architectural facades, they investigated self-cleaning facade paints over an 8-year period in polluted areas of Cairo, Egypt, and Beirut, Lebanon. Their proposed method can serve as an economical solution for pollution control in developing countries, preventing unnecessary expenses and saving time [57]. Furthermore, Atwal et al. highlighted the widespread use of photo catalysis in the construction industry. They discussed the successful cost reduction in maintaining photocatalytic membranes at Tokyo Narita International Airport during its reconstruction in 2006. Additionally, Zahra Gholami et al. examined the cleaning costs associated with the Imam Ali Boulevard facade in Tehran over a 10-year period. Their results demonstrated that self-cleaning nanomaterials significantly reduced the need for facade cleaning and repainting, ultimately contributing to an improved urban environment [20]. Ana Rabaićik et al. investigated the impact of photocatalytic self-cleaning processes on the reducing air pollutants harmful to human health. Their findings suggest that photo catalysis can effectively reduce air pollution and extend the lifespan of buildings [18]. Mahmoudi et al. analyzed the role of nanotechnology in developing sustainable building materials. They discussed the potential of nanotechnology to reduce energy consumption and improve air quality in residential and commercial buildings [5]. Akbari et al. explored the importance of energy optimization in buildings and the potential of nanotechnology to address these challenges. They discussed how nanotechnology can lead to the development of new materials, tools, and systems with enhanced properties and functions at the molecular and atomic levels, contributing to reduced energy consumption and environmental pollution [25]. The integration of nanomaterials in building design not only contributes to energy efficiency but also presents certain security considerations. For instance, the use of self-cleaning nanomaterials can reduce maintenance costs and risks associated with high-rise cleaning operations. However, it is crucial to evaluate the long-term durability and potential environmental impacts of these materials to ensure they do not compromise building safety or occupant health [58].

According to previous research, photocatalytic materials exhibit high albedo properties, enabling them to reflect infrared radiation and maintain ambient temperature and stability under high temperatures [59]. Challenge of using cool materials (high albedo) and maintaining high albedo on urban surfaces is enormous [60]. Tang et al. study shows the properties using photocatalytic materials with high albedo, including self-cleaning capabilities and cooling properties, can reduce peak energy demand and mitigation of the urban heat island effect on roofs and walls. The energy-saving effect of cool materials has been widely studied through experimental measurement and building energy simulation methods [4]. Zahra Gholami et al. investigated thermal comfort in self-cleaning materials on the facade of Jolfa Square in Isfahan. They concluded that the use of high-albedo cooling materials modified with titanium dioxide is not only effective in self-cleaning but also has a significant impact on reducing urban heat [16]. Dias, Andrade et al. found that using white coatings in paints or other building materials can increase infrared reflectance on building facades [61]. Wang et al. further noted that the use of white coatings based on titanium dioxide and zinc oxide can effectively reduce costs and improve thermal stability in architectural designs. White coatings filled with TiO_2_ and ZnO are widely used in architectural coatings due to their low cost and high thermal stability. To maintain near-infrared reflectance of the coatings, a hydrophobic surface can provide anti-fouling and self-cleaning properties [62]. High energy consumption and air pollution and its other consequences have a direct negative impact on the potential of continuous monitoring of long-term human health, in this regard, Several trends in computing and communications technology have converged to advance continuous health monitoring from a distant vision to the verge of practical feasibility [63]. In the context of smart building technologies, the implementation of lightweight cryptography is essential for ensuring secure data transmission between sensors and control systems. Lightweight cryptographic algorithms are designed to provide robust security with minimal computational overhead, making them ideal for resource-constrained environments like IoT-enabled smart buildings. Related works by researchers such as Louie et al. have demonstrated the effectiveness of lightweight cryptographic solutions in safeguarding data integrity and privacy in smart infrastructure [64,65].

Previous research in the field of optimal form-finding has primarily focused on either solar radiation or energy consumption. However, limited investigations have explored the combination of these two critical aspects. Specifically, the integration of novel materials, such as self-cleaning nanomaterials, into architectural design for optimal form-finding considering both energy efficiency and solar exposure remains largely unexplored. Therefore, this multidimensional study aims to address this gap by examining three key parameters: climatic form optimization, vertical solar radiation on building surfaces, and the impact of cutting-edge nanomaterial technology. Additionally, the research employs a hybrid approach, combining quantitative modeling and qualitative simulation methods. The spatiotemporal context of this study further emphasizes the importance of aligning modular design patterns with regional energy consumption. By selecting a superior climatic modular pattern, this research identifies an energy-conscious form that also accounts for the influence of self-cleaning nanomaterials.

## 3 Material & method

This research aims to determine the optimal building form and orientation for an educational building located in Tehran, Iran. The study integrates theoretical foundations with the specific climatic conditions of the site to achieve desirable solar radiation reception, reduced energy consumption, and explore the potential benefits of self-cleaning nanomaterials. Tehran, the capital of Iran, is situated in southwestern Asia with a semi-arid climate. Located at approximately 1,190 m above sea level with geographical coordinates of 35°41′N and 51°19′E, the city experiences an average annual precipitation of 220 mm and an average annual temperature of 16.4 °C. Three key factors influence Tehran's climate: The Alborz Mountain range in the north, prevailing westerly winds which bring the rain, and, the desert in the south. With an average relative humidity of 39.7 %, the minimum and average annual temperatures of Tehran are 2.4 °C and 17.4 °C, respectively [27]. The case study focuses on an educational building in the city zone4, at the northeastern part of Tehran. The rectangular-shaped site measures 75 m in width and 130 m in length, covering an area of 9,750 square meters. The proposed building is a three-story concrete structure (Table 3a).

**Table 3:**
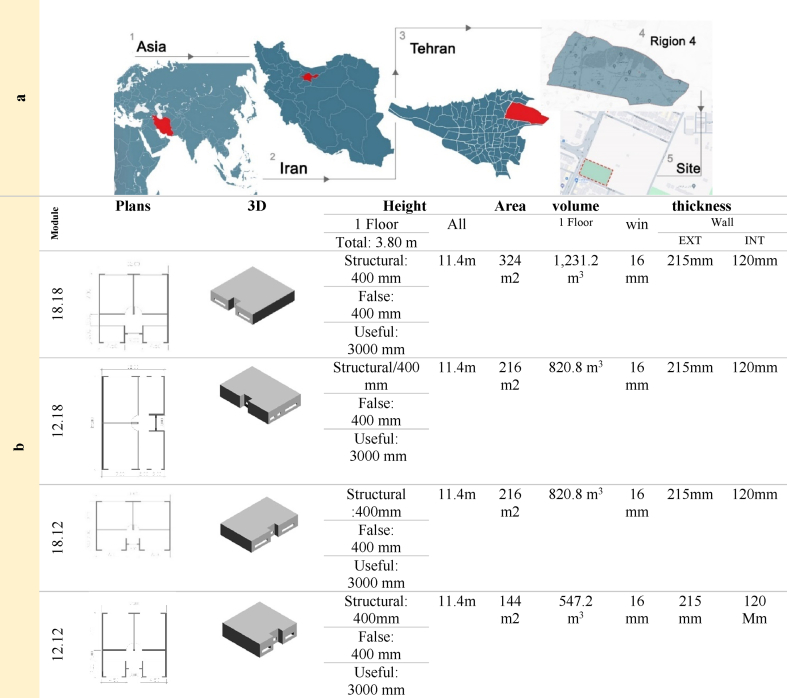
a) Site location b) Specifications of the modules.

**Table 4:** a) Modul orientations b) Materials c) Suggested modular arrangement d) Selected modular arrangement.

A						
**B**	**Name**		**Materials**	**Thickness (mm)**	**Overall**	**U-value**
	External Wall		Microcement	3	215 mm	
			White cement plaster	5		_
			Cement lining coating/1:3	1		
			Cement blocks	20		5.216
			plaster and soil	3		
			Final plaster	2		8.421
			Primer & color	1		
	Window		Frame	4	16 mm	_
			Internal glass	4		5.282
			air between	6		5.553
			External glass	6		5.282
	Door		Wooden body	40	40 mm	
			Glass	6		5.7
			Metal handle	2		_
	Ceiling	Floor	Epoxy flooring	4	54 mm	
			Light weight concrete	50		5.216
		Structural	concrete slab	400	460	9.596
			plaster and soil	3		–
			Final plaster	2		8.421
			Primer & color	1		
**C**				d

**Table 5:** a) Per capita energy consumption on the coldest and hottest day in 4 modules b) The difference of energy consumption in the modular arrangement of 4 types with & without nanomaterials.

		**energy**	**Time** **Day**	**06:00**	**07:00**	**08:00**	**09:00**	**10:00**	**11:00**	**12:00**	**13:00**	**14:00**	**15:00**	**16:00**	**17:00**	**18:00**
a	Modul1(12.12) S = 144 m^2^	EC	Coldest day	7.73	7.94	7.97	7.26	6.26	5.17	4.13	3.30	2.79	2.58	2.62	2.93	3.58
			Hottest day	0.00	0.00	0.00	0.00	0.00	0.35	1.26	2.18	2.92	3.43	3.70	3.77	3.68
		EC per capta	Coldest day	0.0536	0.0551	0.0553	0.0504	0.0434	0.0359	0.0286	0.0229	0.0193	0.0179	0.0181	0.0203	0.0248
			Hottest day	0.00	0.00	0.00	0.00	0.00	0.0024	0.0087	0.0151	0.0202	0.0238	0.0256	0.0261	0.0255
	Module (12.18)S = 216 m^2^	EC	Coldest day	10.98	11.27	11.33	10.55	9.38	8.02	6.66	5.49	4.68	4.26	4.27	4.68	5.48
			Hottest day	0.00	0.00	0.00	0.00	0.00	0.21	1.33	2.55	3.57	4.32	4.75	4.91	4.77
		EC per capta	Coldest day	0.0508	0.0521	0.0524	0.0488	0.0434	0.0371	0.0308	0.0254	0.0216	0.0197	0.0197	0.0216	0.0253
			Hottest day	0.00	0.00	0.00	0.00	0.00	0.0009	0.0061	0.0118	0.0165	0.02	0.0219	0.0227	0.0220
	Module 3 (18.12)S = 216 m^2^	EC	Coldest day	11.26	11.57	11.61	10.55	9.1	7.52	6.03	4.83	4.12	3.82	3.89	4.35	5.31
			Hottest day	0.00	0.00	0.00	0.00	0.00	0.44	1.78	3.08	4.13	4.87	5.23	5.33	5.19
		EC per capta	Coldest day	0.0521	0.0535	0.0537	0.0488	0.0421	0.0348	0.0279	0.0223	0.0190	0.0176	0.0180	0.0201	0.0245
			Hottest day	0.00	0.00	0.00	0.00	0.00	0.0020	0.0082	0.0142	0.0191	0.0225	0.0242	0.0246	0.0240
	Module 4 (18.18)S = 324 m^2^	EC	Coldest day	15.98	16.4	16.49	15.32	13.6	11.64	9.69	8.01	6.86	6.28	6.31	6.93	8.11
			Hottest day	0.00	0.00	0.00	0.00	0.00	0.29	1.89	3.6	5.05	6.11	6.71	6.9	6.66
		EC per capta	Coldest day	0.0493	0.0506	0.0508	0.0472	0.0419	0.0359	0.0299	0.0247	0.0211	0.0193	0.0194	0.0213	0.0250
			Hottest day	0.00	0.00	0.00	0.00	0.00	0.0008	0.0058	0.0111	0.0155	0.0188	0.0207	0.0212	0.0205
b	Type 1 (12.12)		Coldest day	6.51	6.7	6.81	6.56	5.98	5.05	3.92	2.82	2.73	3.07	2.73	2.26	2.22
			Hottest day	0	0	0	0.26	0.7	1.07	1.41	1.7	1.96	2.27	2.67	3.08	3.43
	Type 2 (12.18)		Coldest day	6.41	6.6	6.75	6.65	6.21	5.34	4.2	3.37	2.98	2.89	2.45	2.01	2.54
			Hottest day	0	0	0	0	0.52	1.76	1.32	1.63	1.95	2.31	2.66	2.97	3.22
	Type 3 (18.12)		Coldest day	6.43	6.63	6.75	6.54	5.97	4.94	3.56	3.03	3.46	3.15	2.61	2.08	2.11
			Hottest day	0	0	0	0	0.6	0.79	1.11	1.47	1.79	2.11	2.49	2.82	3.09
	Type 4 (18.18)		Coldest day	6.57	6.76	6.86	6.6	6.01	5.03	3.86	2.72	2.69	3.02	2.72	2.23	2.32
			Hottest day	0	0	0	0	0.95	1.2	1.59	1.9	2.13	2.42	2.77	3.14	3.44

EC per capta: energy consumption divided by module area (kW/h m^2^)/EC: Energy consumption (kW/h).

This study combines two research methods: quantitative modeling and qualitative simulation. Modeling was performed using Rhino software, while simulation utilized Energy Plus software, which allows calculating the effects of various heating, cooling, ventilation, lighting, and window systems and is widely used by researchers to evaluate building energy consumption and achieve net-zero energy. In Energy Plus software, algorithms such as genetic algorithms are used for optimization. However, in the present study, sample comparisons have been made using comparative shape analysis because the research focuses on the Nano technique's impact on energy consumption.

**Fig. 1:**
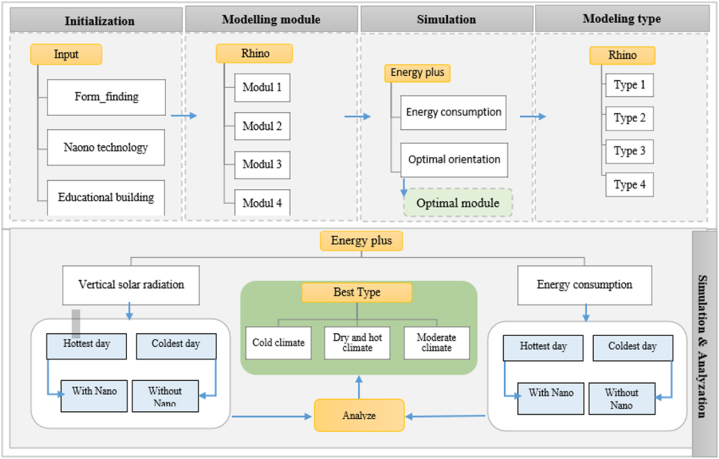
Work flow.

**Fig. 2:**
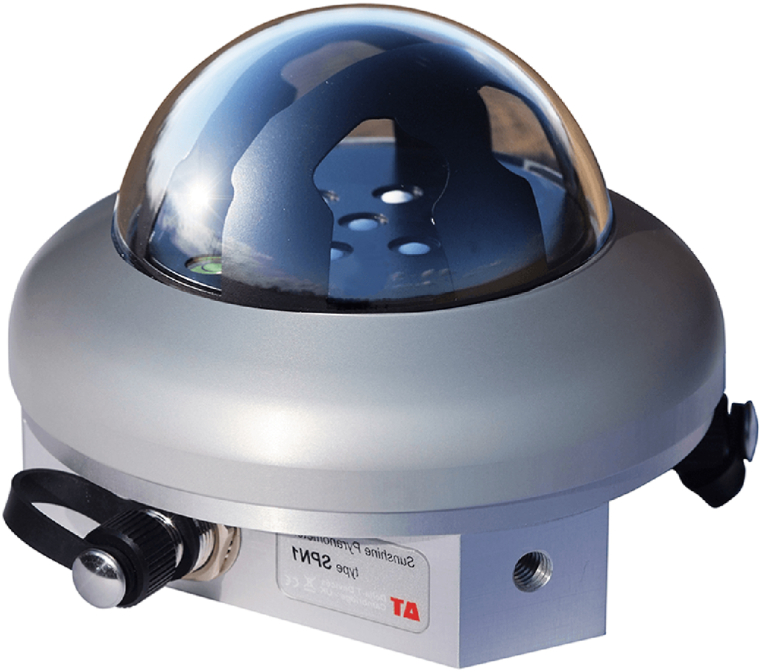
SPN1 Sunshine Pyranometer.

Several optimal designs have been identified along the research path. The research stages include the following (Fig. 1):

1. A comprehensive review of existing literature was conducted to identify relevant research on optimal form-finding, educational buildings, and self-cleaning nanomaterials.
2. Based on the literature review, four modular classroom layouts were proposed, each maintaining a 2:3 aspect ratio (x: y configurations explored included x = y, x < y, and x > y). Each module comprises two classrooms, a connecting corridor, and an interactive space. A constant window-to-wall ratio (WWR) of 25 % was applied to all facades of each module (Table 4b).
3. Energy consumption per capita and optimal orientation for the modules were calculated for the coldest day (February 4th) and the hottest day (July 4th) of the year in Tehran. The most energy-efficient module orientation was identified.
4. The average annual energy consumption in Tehran was considered to determine the city-specific optimal orientation. The chosen optimal model exhibits a south orientation with a range of −30 to +30° relative to south, incorporating a ten-degree offset for simulation and desired final orientation (Table 4a).
5. To determine the optimal final form, fifteen modular layouts were proposed (Table 4c). Of these, only four layouts were evaluated as suitable for the educational building design (Table 4d). Based on the established architectural and programmatic standards, each of these layouts comprises nine optimized modules arranged over three floors. The selection of these layouts was guided by the feasibility of design and the achievement of the minimum required area for the educational space.
6. Energy consumption and radiation simulations were conducted on the vertical surfaces of the four selected modular layouts for the 2 aforementioned days.
7. The impact of incorporating self-cleaning nanomaterials, used in the building façade, on the energy consumption and building surface radiation was evaluated for both days. Then, compared and analyzed with the results of the previous step (Table 5). The TiO_2_ nanomaterial (Nano white color) employed for all exterior vertical surfaces exhibits a reflectance coefficient ranging from 0.7 to 0.9 and an albedo of 0.9.
8. The optimal modular arrangement for the Tehran climate and suitable façades for the application of self-cleaning nanomaterial paint were identified.
9. The validity of this research was confirmed by comparing the results of simulation and measurements. The amount of radiation on the horizontal surface per square meter was measured at 12:00 p.m. on May 11th using a SPN1 Sunshine Pyranometer (Fig. 2). Subsequently, this radiation level was simulated using Energy Plus software. The measured amount and the simulation were 964 W/m^2^ and 963.68 W/m^2^, respectively. The difference between these two values was 0.32, indicating the credibility of Energy Plus software in the field of radiation. Energy Plus software is widely used by researchers to assess building energy consumption. It serves as an energy analysis and thermal load simulation tool, allowing simulation of building performance related to lighting, daylighting, water heating, and on-site energy production. It has been validated as an effective tool for conducting simulations [57].
10. The findings of this study highlight the effectiveness of specific modular designs and orientations in reducing energy consumption and managing solar radiation. However, further investigation is needed to understand the long-term impact of self-cleaning nanomaterials, which could potentially enhance energy savings. The limitations of this project include the inability to test on existing buildings and the lack of data on the heat transfer properties of nanomaterials.

## 4 Findings

Leveraging nanotechnology, this study determined the optimal module, orientation and layout to reduce energy consumption of an educational building in Tehran. In addition to energy consumption, radiation on vertical surfaces was also simulated and analyzed.

### 4.1 Optimal module and orientation

**Fig. 3:**
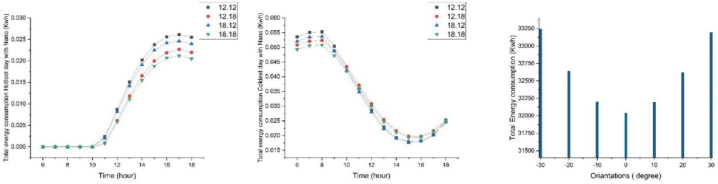
Annual energy consumption of module 18.18 at angles of −30 to +30 (right), comparison of per capita energy consumption of 4 modules with nanomaterials on the coldest day (middle) and the hottest day (left).

Energy consumption simulations were conducted for the four proposed modules to determine the most suitable climate-responsive module throughout the year's coldest and hottest days, from sunrise to sunset. On the coldest day, energy consumption took a gradual upward trend in the morning (6–8 a.m.), followed by a decline near noon, reaching its minimum at 2–4 p.m. On the hottest day, energy consumption remained constant from 6 to 10 a.m., followed by an upward trend near noon, reaching its maximum at 3–5 p.m., and then decreasing slightly until sunset. Based on the energy consumption simulations, the optimal module dimensions were determined to be 18 0.18 m. To further reduce energy consumption, an analysis was conducted to identify the optimal building orientation based on annual energy consumption, considering angles between −30 and + 30° to the south (Fig. 3).

### 4.2 Optimal arrangement

**Fig. 4:**
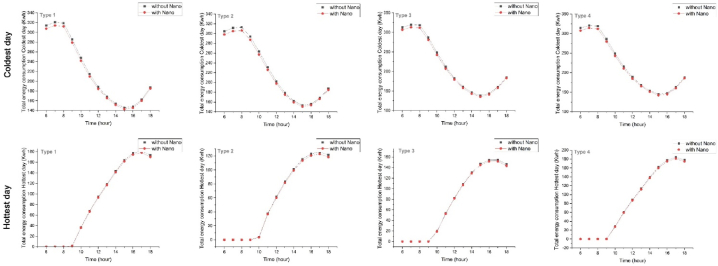
The amount of energy consumption of four types on the coldest day (up) and the hottest day (down) with Self-cleaning nanomaterials.

In determining the optimal climate-responsive layout, in addition to energy consumption, radiation on vertical surfaces was also simulated. The optimal module area (18m.18m) is 324 square meters. According to national building regulations, the maximum permissible land occupancy is 50 %. Therefore, nine modules with an area of 2916 square meters per floor were considered (Fig. 4d).

#### 4.2.1 Energy consumption and module arrangements

Energy consumption patterns were analyzed with and without nanotechnology for the three proposed module arrangement types. The results of Type1 energy consumption analysis showed a gradual upward trend from 6 to 7 a.m. in the coldest day of the year, followed by a slight decline near 9 a.m., reaching its minimum by 3 p.m. and then, increasing again until sunset. This process remained constant from 6 to 9 a.m. on hottest day, followed by an upward trend until 5 p.m., which decreased gradually until sunset. Furthermore, Type2 energy consumption analysis, on the coldest day of the year, showed an upward trend from 6 to 8 a.m., reaching its maximum at 8 a.m., followed by a downward trend to its minimum by 3 p.m. and then, increasing again until sunset. The consumption remained constant from 6 to 9 a.m. on the hottest day, followed by a slight upward trend until 10 a.m., reaching its maximum by 5 p.m., and then, decreasing until sunset. Energy consumption analysis of Type3 shows a gradual upward trend from 6 to 7 a.m. in the coldest day of the year, followed by a slight decline near 9 a.m., reaching its minimum by 3 p.m., and then, increasing again until sunset. On the hottest day, Energy consumption of Type3 remained constant from 6 to 9 a.m., followed by an upward trend until 4 p.m., and, decreased until sunset. Additionally, energy consumption of Type4 took a gradual upward trend from 6 to 7 a.m. on the coldest day of the year, followed by a slight decline near 9 a.m., reaching its minimum by 3 p.m. and then, increasing again until sunset. The hottest day on the other hand had a constant energy consumption trend from 6 to 9 a.m., followed by an upward trend and reaching its maximum at 5 p.m., and then, decreasing until sunset (Fig. 4).

#### 4.2.2 Solar radiation evaluation of vertical surfaces

**Fig. 5:**
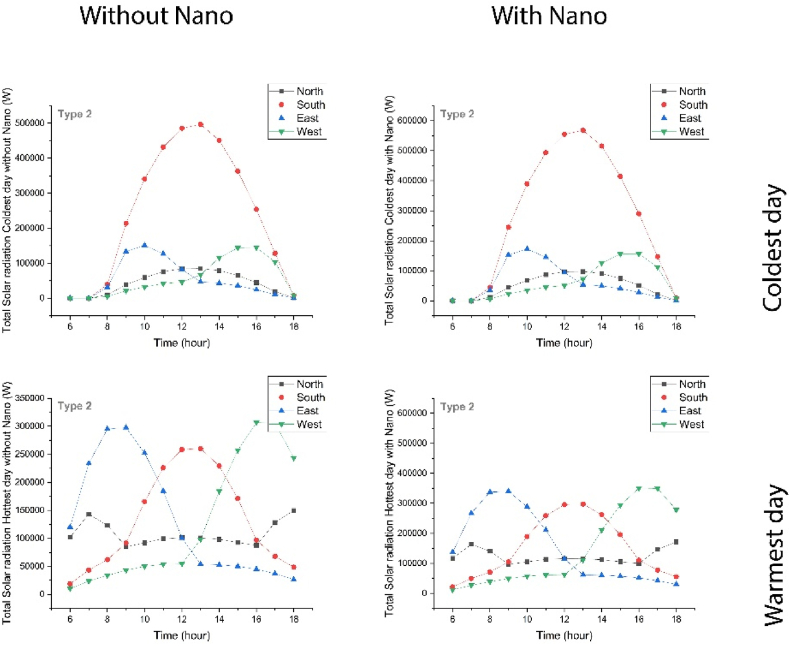
The amount of solar radiation of type 2 on the coldest day (up) and the hottest day (down) with Nano (right) and without Nano (left).

The comparative analysis was conducted to evaluate solar radiation on vertical surfaces with and without the nanotechnology. Results revealed the radiation pattern of the Type1 Eastern Façades to be constant at zero from sunrise until 6 a.m. in the coldest day of the year, reaching its maximum at 10 a.m., and then gradually decreasing to zero by sunset. Furthermore, solar radiation took an upward trend from 6 a.m. on the hottest day of the year, reaching its maximum at 8–10 a.m., and then decreasing until sunset. Solar radiation of the coldest day in Type 2 remained constant at zero from sunrise until 7 a.m., reached its maximum by 10 a.m., and then, gradually decreased to zero by sunset. On the hottest day of the year, solar radiation also took an upward trend from 6 a.m., reached a maximum at 9 a.m., and then decreased until sunset. However, radiation pattern of Type 3 remained constant at zero from sunrise until 7 a.m. on the coldest day of the year, followed by an upward trend, reaching its maximum at 10 a.m., and then decreasing until sunset. On Hottest Day, the pattern took an upward trend until noon, reached its maximum at 8–9 a.m., and then decreased until sunset. Additionally, radiation pattern of Type 4 also remained constant at zero from sunrise until 7 a.m. of the coldest day of the year, followed by an upward trend, reaching its maximum at 10 a.m., and then decreased until sunset. On the hottest day, the pattern took an upward trend from sunrise until noon, reached its maximum at 10 a.m., and then decreased until sunset. For all types, simulation has been done and a sample has been taken, to see the rest, refer to the supplementary. (Fig. 5).

**Fig. 6:**
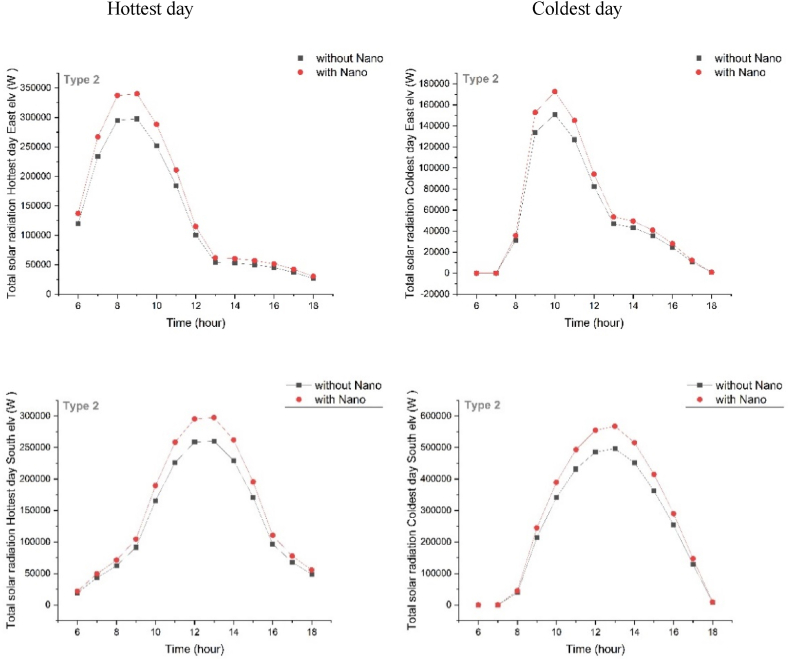
The amount of solar radiation in the east (up) and south (down) Elves of type 2 with Nano on the coldest day (right) and the hottest day (left).

The radiation pattern remained constant at zero from sunrise until 7 a.m. of the coldest day of the year in the southern facades of Type1. Followed by an upward trend, the pattern reached its maximum at 1 p.m., and then, decreased until sunset. On the hottest day of the year, minimum solar radiation was evaluated to be at sunrise, followed by an upward trend until noon, it reached its maximum at 12–1 p.m., and then, decreased gradually by sunset. Similarly, Type2 remained constant at zero from sunrise until 7 a.m. on the coldest day of the year, followed by an upward trend, reaching its maximum at 1 p.m., and then, decreased until sunset. On the hottest day, the radiation pattern took an upward trend from sunrise until noon, reaching its maximum at 12–1 p.m., and then, decreased until sunset. In Type 3, the pattern also remained constant at zero from sunrise until 7 a.m., followed by an upward trend, reaching its maximum at 1 p.m., and then, decreased until the sunset of the coldest day of the year. On the hottest day of the year, radiation pattern took an upward trend from 6 a.m., reaching its maximum at 1 p.m., and then, decreased until sunset. On the coldest day of the year, Type4 radiation pattern remained constant at zero from sunrise until 7 a.m., followed by an upward trend, reaching its maximum at 1 p.m., and then decreasing gradually until sunset. On the hottest day of the year, the radiation pattern took an upward trend from 6 a.m., reaching its maximum at 12–1 p.m., and then, decreasing gradually by sunset. By applying nanotechnology, radiation level increased in all of the four studied types on both the coldest and the hottest day of the year. The maximum increase was observed on the east and south facades. Notably, Nanotechnology affected radiation in the hottest day. As the amount of radiation decreased on surfaces, it prevented the undesirable heat from entering the building. However, the building would become more climate-sensitive as this amount increases on the coldest day of the year. For all types, simulation has been done and a sample has been taken, to see the rest, refer to the supplementary (Fig. 6).

### 4.3 Arrangement comparison of four types

**Fig. 7:**
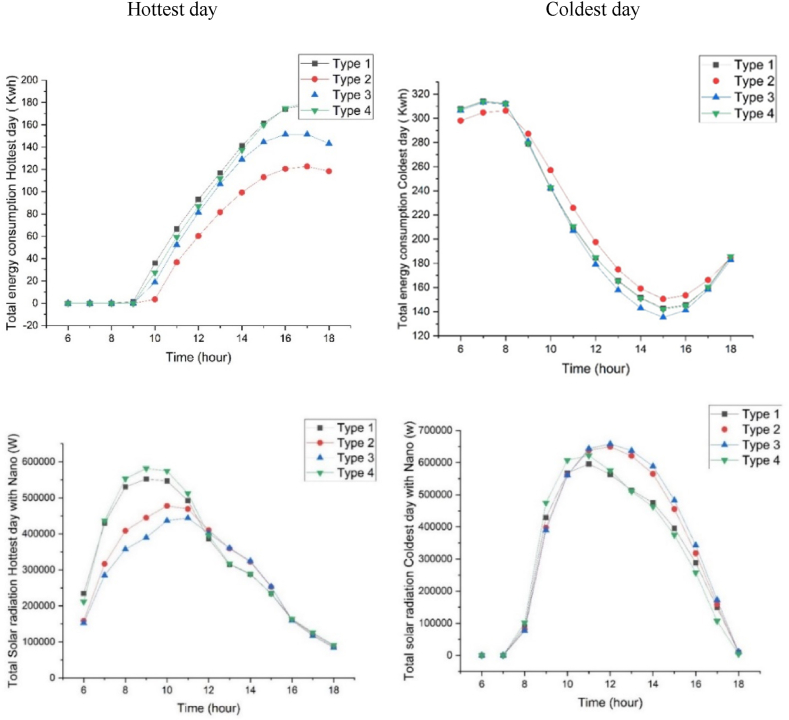
Comparison of the amount of energy consumption (top) and the amount of solar radiation (down) of 4 types with nanomaterials on the coldest day (right) and the hottest day (left).

By comparing the effect of self-cleaning nanomaterials on energy consumption during the coldest and the hottest day of the year, it was determined that Type3 and Type2 coatings had lower energy consumption, respectively. The results showed that energy consumption is deficient, during the cold daytime and warm morning. Additionally, applying nanotechnology increased the received radiation on the east and south-facing surfaces on both mentioned days (Fig. 7). On the coldest day, the received radiation remained constant from sunrise until 7 a.m., then increased, reaching its maximum at 12 noon, followed by a decrease until sunset. Similarly, on the hottest day, the received solar radiation started at its minimum level at sunrise, increased until 9 a.m., and then, decreased until sunset. Analyzing the graphs revealed a noticeable difference in radiation levels between east and south-facing surfaces for all four types between 10 a.m. and 1 p.m. on the coldest day of the year, and, between 8 a.m. and 11 a.m. on the hottest day of the year.

Type3 had the highest radiation reception at 12 p.m. on the coldest day, and is the most suitable proposed pattern for cold climates, as it receives the maximum solar radiation. In the second place, Type4 was the type with the best radiation reception during winter time due to capturing the most desirable morning radiation. Additionally, on the hottest day, Type3 had the lowest radiation reception and is the most suitable proposed pattern for warm climates. Type4 also receives favorable morning radiation on this day, making it compatible with all climates except for the cold climate. Results show that Types1 and Type2 are desirable for moderate climates due to their reception of intermediate radiation (Fig. 7).

## 5 Discussion

This study simulated and analyzed energy consumption and radiation in the studied sample to investigate the climatic impact of self-cleaning nanotechnology. The results were reviewed in three sections: determining the optimal modulation and orientation, determining the optimal arrangement, and, comparing the arrangement of all four studied types after applying self-cleaning nanomaterials.

### 5.1 Optimal module and orientation

By analyzing the graphs of energy consumption patterns for the four proposed modules, the module with dimensions of 18.18m × 18.18m has the lowest energy consumption per unit area (energy consumption divided by module area) despite having the highest overall energy consumption (Table 5a). Additionally, the building orientation of 0° (north-south) resulted in the lowest annual energy consumption, while an orientation of 30° resulted in the highest (Table 7). Therefore, this study evaluates the north-south orientation to be the optimal orientation for minimizing energy consumption (Fig. 3).

**Table 6:** Radiation on the south façade (without nanomaterial) in the coldest and the hottest day of the year.

Day		Coldest day				Hottest day			
Time	Nano	Type 1	Type 2	Type 3	Type 4	Type 1	Type 2	Type 3	Type 4
11:00	without Nano	325904.72	431630.96	445793.28	338042.38	172369.82	226108.36	233399.94	174429.36
	with Nano	372462.54	493292.53	372266.01	386334.14	196994.09	258409.56	266742.78	199347.84
12:00	without Nano	368574.47	485625.46	501344.06	373322.92	196775.20	258523.97	266836.32	197251.90
	with Nano	421227.96	555000.52	411318.50	426654.76	224885.94	295455.97	304955.80	225430.74
13:00	without Nano	381150.17	496694.11	516310.96	376188.40	199241.58	260367.78	269849.48	197395.79
	with Nano	435600.20	567650.41	417749.48	429929.60	227704.67	297563.18	308399.41	225595.18
14:00	without Nano	352264.68	451112.39	477062.21	339828.77	177142.42	229271.06	239202.17	174086.94

**Table 7:** The radiation difference in the Eastern and Southern facades with Nano on the coldest and hottest days of the year.

		6:00	7:00	8:00	9:00	10:00	11:00	12:00	13:00	14:00	15:00	16:00	17:00	18:00
Coldest day	Type 1	0	0	11218.67	53637.61	70,978.2	74,490.4	70,414.4	64,265.5	59,411.5	49,477	36,027.5	18,668.4	1208.318
	Type 2	0	0	10,054.8	49,708.2	70,250.9	79,808.4	81,149.9	77,642.4	70,630.2	56,941.4	39,771.8	19,876.3	1,234.19
	Type 3	0	0	9,580.07	48,716.75	70,130.67	70,444.3	82,277.17	79,647.99	73,604.5	60,374.74	42,818.63	21,608.89	1,391.72
	Type 4	0	0	12,754.1	59,372.1	75,895.2	77,733.1	71,890.5	63,823.8	57,874.9	46,870.3	32,215.3	13,392.24	548.43
Hottest day	Type 1	29,304.2	53,750.5	66,316.2	69,011.5	68,401.2	61,550.3	48,318.7	39,370.4	36,020.6	29,283.2	20,250.1	15,490.5	11,182.32
	Type 2	19,849.9	39,579.9	51,052.6	55,606	59,713.9	58,646.1	51,310.4	44,926.4	40,312.2	31,598	20,279	15,013.7	10,736.82
	Type 3	19,068.6	35,645.6	44,693.5	48,722.7	54,572.7	55,498.5	50,244.2	45,094.2	40,600.5	31,707.2	19,937.3	14,656.7	10,521.96
	Type 4	26,440.9	54,574.8	69,122.8	72,688.4	71,789.8	64,058.8	49,523.1	39,628.9	36,066.2	29,329.4	20,526.4	15,771.7	11,366.44

### 5.2 Optimal arrangement

The maximum energy consumption occurred during cold days between 6 a.m. and 9 a.m., while during hot days, it was between 4 p.m. and 6 p.m. (Table 5). Additionally, the highest radiation exposure on east-facing surfaces occurred during the coldest day between 9 a.m. and 11 a.m., and during the hottest day between 8 a.m. and 10 a.m. (Supplementary table). Furthermore, the amount of radiation was calculated at all times, but the south-facing surfaces received the most radiation during both selected days, peaking between 11 a.m., and 2 p.m. (Table 6).

### 5.3 Arrangement comparison of four studied types

By analyzing and comparing the difference in energy consumption before and after applying nanomaterials for all of the four types, it was evident that energy consumption decreased on both selected days. Since the morning of the hottest day, requires less cooling and heating and only necessitates lighting, there was no significant difference in energy consumption with or without nanotechnology. However, due to increased cooling needs in the afternoon, the difference in energy consumption became more pronounced when using nanotechnology. In contrast, on the coldest day of the year, the most significant difference occurred in the morning, indicating higher heating consumption (Table 5). The maximum difference in energy consumption between with Nano and without Nano types was 6.86 kW-hours at 8 a.m. of the coldest day, and 3.44 kW-hours at 6 p.m. of the hottest day (Table 5). Additionally, comparing the radiation difference between east and south-facing vertical surfaces for all four types on the coldest and the hottest days of the year revealed that despite using self-cleaning nanomaterials, the maximum radiation increased between 10 a.m. and 1 p.m. in the winter season, and, between 8 a.m. and 11 a.m. in the summer season (Table 7). The highest radiation difference between with Nano and without Nano types was in Type 3 (560,595.4 Watts) on the coldest day of the year, highlighting the significant impact of nanotechnology in this type. Furthermore, Type 3 also had the lowest radiation difference on the hottest day, representing as the most climate-sensitive type. Also, the maximum radiation difference on vertical surfaces between Nano and non-Nano types was 82,277.17 Watts at 12 p.m. of the coldest day and 72,688.4 Watts at 9 a.m. of the hottest day.

## 6 Conclusion

Considering the significance of climate in architectural design, in most previous research, comprehensive investigations with the specified objectives for climatic form finding, energy consumption, solar radiation and Also, the utilization of innovative technologies and materials such as self-cleaning nanomaterials were not found. The distinctive feature of this research is to achieve the comprehensive and multifaceted goal of climate design of the building in integration with the use of new materials for educational buildings. Among the four proposed modular class arrangements, the optimal module for semi-arid climates was determined as 18.18m with the orientation of 0° (North-South) based on energy consumption. Also, by comparing the effect of color in self-cleaning nanomaterials on the energy consumption of the four arrangements, this paper reveals Type 3 as the best arrangement in the coldest day of the year analysis. This is while Type 2 performed optimally in the hottest day. Further simulation analysis of energy consumption and radiation on vertical surfaces for the hottest and the coldest days of the year across four modular educational building arrangements introduces Type 3 as the most suitable option for the cold seasons. Similarly, on the hottest day of the year, Type 3 also had the lowest radiation reception, making it the most climate-sensitive option for the warm seasons.

Investigating the impact of self-cleaning nanomaterials on energy consumption and solar radiation revealed that the application of nanotechnology increases the maximum received radiation on south and east-facing surfaces for all four types on both selected days. Analysis shows that nanotechnology reduces direct sunlight radiation in the hottest day of the year, preventing excessive heat gain in buildings. Based on the comparison of received solar radiation analysis after implementing nanotechnology, Type 3 was, again, recognized as the most suitable type for the coldest day of the year. Similarly, this arrangement type is the selected option for the warm seasons due to its lower radiation exposure and optimal shading.

Comparing the difference of energy consumption before and after applying nanotechnology, showed that the energy consumption decreases on both the hottest and the coldest days of the year. The total daily radiation difference between Nano and non-Nano types was found to be the highest in the Type 3 arrangement (560,595.4 Watts) on the coldest day of the year, highlighting the greater impact of nanotechnology on this type.

Based on the final results of comparing the energy consumption and radiation on vertical surfaces before and after the implementation of selected nanotechnology, it could be concluded that Type 3 is representing the best building arrangement in terms of energy consumption and solar radiation in semi-arid climates. It is also important to note that while energy consumption for all arrangements decreased in both the coldest and the hottest days of the year after implementing the chosen nanotechnology, the radiation on vertical surfaces increased before and after nanotechnology application for all four arrangement types. Hence, the results of this research reveal that while the use of this technology is favorable in cold seasons, it is unfavorable for hot seasons. Considering that seasonal change of facade materials is impractical, employing this technology in the city of Tehran with semi-arid climate is recommended for the cooler sub-climatic zones of the city, including the northern foothills. Furthermore, results show that the application of this technology is a regional priority for the western and southwestern facades in Tehran.

Integrating self-cleaning nanomaterials in architecture enhances energy efficiency, occupant comfort, and reduces maintenance costs. These materials optimize passive solar heating, natural ventilation, and shading, while repelling dirt and pollutants. The result is sustainable, aesthetically pleasing, and low-maintenance buildings.

The future research with the proposed title aims to explore the following directions:

1. Investigate various nanomaterials (such as graphene, silver nanoparticles, and TiO₂-based nano coatings) and their suitability for enhancing energy efficiency in building design.
2. Explore how these materials can be integrated into building components (e.g., façades, windows, and insulation) to optimize energy performance.
3. Utilize advanced simulation tools to model the impact of nanomaterials on energy consumption and solar radiation.
4. Consider different orientations, configurations, and layouts of educational buildings to identify optimal designs.

## CRediT authorship contribution statement

**Hannaneh Asgari:** Writing – review & editing, Writing – original draft, Software, Methodology, Investigation, Funding acquisition, Formal analysis, Data curation, Conceptualization. **Samaneh Taghdir:** Writing – review & editing, Writing – original draft, Validation, Supervision, Resources, Project administration, Methodology, Investigation, Funding acquisition, Data curation, Conceptualization. **Rezvaneh Amrollahi:** Writing – review & editing, Writing – original draft, Visualization, Validation, Supervision, Project administration, Methodology, Investigation, Data curation, Conceptualization. **Zahra Barzegar:** Writing – review & editing, Writing – original draft, Validation, Supervision, Software, Resources, Methodology, Investigation, Funding acquisition, Data curation, Conceptualization.

## Data availability

Some data have been directly included in the article, while additional data can be obtained upon reasonable request from the corresponding author.

## Declaration of competing interest

The authors declare that they have no known competing financial interests or personal relationships that could have appeared to influence the work reported in this paper.
