# Supplementary material for: The impact of nanomaterials on energy-centric form-finding of educational buildings in semi-arid climate
Source: Heliyon. 2024 Nov 4;10(21):e39882. doi: 10.1016/j.heliyon.2024.e39882 (PMC11577218; doi:10.1016/j.heliyon.2024.e39882)
Supplement: Multimedia component 1 [file mmc1.docx]

**The impact of Nanomaterials on energy-centric form-finding of educational buildings in semi-Arid climate**

Hannaneh Asgari^1^, Samaneh Taghdir1, Rezvaneh Amrollahi2^,*^, Zahra Barzegar 3

^1^ School of Architecture and Environmental Design, Iran University of Science a and Technology,, Tehran, 16846, Iran

2 School of Physics Iran University of Science a and Technology, , Tehran, 16846, Iran

3Director of Environmental Studies, Tehran Urban Research and Planning Centre(TURPC),Tehran,1964635611, Iran

*[Amrollahir@iust.ac.ir](mailto:Amrollahir@iust.ac.ir)

**Supplementary**

**Radiation on the East Elves on the coldest and hottest day in 4 types with nanomaterials.**

| Hottest day | | | | Coldest day | | | | Day | |
| --- | --- | --- | --- | --- | --- | --- | --- | --- | --- |
| Type 4 | **Type 3** | **Type 2** | **Type 1** | **Type 4** | **Type 3** | **Type 2** | **Type 1** |  | **Time** |
| 169653.79 | 113903.07 | 120012.11 | 189838.46 | 0.00 | 0.00 | 0.00 | 0.00 | without Nano | **06:00** |
| 193890.05 | 130174.94 | 137156.70 | 216958.24 | 0.00 | 0.00 | 0.00 | 0.00 | with Nano |  |
| 346746.60 | 204796.82 | 233697.59 | 341328.03 | 0.00 | 0.00 | 0.00 | 0.00 | without Nano | **07:00** |
| 396281.83 | 234053.51 | 267082.96 | 390089.18 | 0.00 | 0.00 | 0.00 | 0.00 | with Nano |  |
| 433890.46 | 248787.65 | 295209.49 | 414646.08 | 54702.76 | 30953.35 | 31269.02 | 51588.91 | without Nano | **08:00** |
| 495874.81 | 284328.74 | 337382.27 | 473881.23 | 62517.44 | 35375.25 | 35736.03 | 58958.75 | with Nano |  |
| 435986.11 | 246695.41 | 297706.19 | 411159.02 | 231797.48 | 131163.61 | 133707.09 | 218606.01 | without Nano | **09:00** |
| 498269.84 | 281937.61 | 340235.65 | 469896.02 | 264911.40 | 149901.26 | 152808.11 | 249835.44 | with Nano |  |
| 372968.90 | 210911.85 | 252213.76 | 351519.75 | 252086.01 | 142903.01 | 150986.15 | 238171.69 | without Nano | **10:00** |
| 426250.17 | 241042.12 | 288244.30 | 401736.86 | 288098.30 | 163317.73 | 172555.60 | 272196.22 | with Nano |  |
| 273982.41 | 155089.21 | 184414.22 | 258482.01 | 206089.17 | 117316.86 | 127027.67 | 195528.10 | without Nano | **11:00** |
| 313122.76 | 177244.81 | 210759.11 | 295408.01 | 235530.48 | 134076.41 | 145174.48 | 223460.68 | with Nano |  |
| 149409.54 | 84873.38 | 115026.99 | 141455.64 | 129910.53 | 74596.14 | 82423.54 | 124326.90 | without Nano | **12:00** |
| 170753.75 | 96998.15 | 100648.62 | 161663.59 | 148469.17 | 85252.73 | 94198.33 | 142087.88 | with Nano |  |
| 80006.05 | 45810.39 | 61847.49 | 76350.66 | 70578.25 | 85252.73 | 46802.81 | 68708.21 | without Nano | **13:00** |
| 91435.49 | 52354.73 | 54116.55 | 87257.89 | 80660.85 | 47114.20 | 53488.92 | 78523.67 | with Nano |  |
| 78376.61 | 45000.95 | 60473.64 | 75001.58 | 65295.90 | 47114.20 | 43298.74 | 63615.47 | without Nano | **14:00** |
| 89573.27 | 51429.66 | 52914.44 | 85716.09 | 74623.89 | 43622.04 | 49484.27 | 72703.39 | with Nano |  |
| 74371.83 | 42868.79 | 57208.28 | 71447.98 | 53968.38 | 41224.93 | 35743.97 | 52631.90 | without Nano | **15:00** |
| 84996.38 | 48992.90 | 50057.25 | 81654.84 | 61678.15 | 36090.44 | 40850.25 | 60150.74 | with Nano |  |
| 67435.03 | 39136.26 | 51609.96 | 65227.10 | 37141.48 | 38169.28 | 24557.54 | 36273.39 | without Nano | **16:00** |
| 77068.60 | 44727.15 | 45158.72 | 74545.26 | 42447.40 | 31579.14 | 28065.77 | 41455.31 | with Nano |  |
| 55678.31 | 32492.61 | 42394.61 | 54154.36 | 16175.63 | 9505.46 | 10662.02 | 15842.44 | without Nano | **17:00** |
| 63632.35 | 37134.42 | 37095.28 | 61890.69 | 18486.43 | 10863.39 | 12185.17 | 18105.64 | with Nano |  |
| 40128.08 | 23522.96 | 26582.60 | 39204.93 | 1083.05 | 638.92 | 710.09 | 1064.87 | without Nano | **18:00** |
| 45860.66 | 26883.38 | 30380.11 | 44805.64 | 1237.77 | 730.20 | 811.53 | 1217.00 | with Nano |  |
